# 4-Bromo­acetyl-3-phenyl­sydnone

**DOI:** 10.1107/S1600536812026049

**Published:** 2012-06-16

**Authors:** Hoong-Kun Fun, Tze Shyang Chia, Nithinchandra, Balakrishna Kalluraya, Shobhitha Shetty

**Affiliations:** a X-ray Crystallography Unit, School of Physics, Universiti Sains Malaysia, 11800 USM, Penang, Malaysia; b Department of Studies in Chemistry, Mangalore University, Mangalagangotri, Mangalore 574 199, India

## Abstract

In the title compound (systematic name: 4-bromoacetyl-1,2,3-oxadiazol-3-ylium-5-olate), C_10_H_7_BrN_2_O_3_, the 1,2,3-oxadiazole ring and bromo­acetyl group are essentially planar [maximum deviation = 0.010 (4) and 0.013 (3) Å respectively] and form dihedral angles of 59.31 (19) and 67.96 (11)°, respectively, with the phenyl ring. The 1,2,3-oxadiazole ring is twisted slightly from the mean plane of the bromo­acetyl group, forming a dihedral angle of 9.16 (24)°. In the crystal, mol­ecules are linked by pairs of weak C—H⋯O hydrogen bonds into inversion dimers with *R*_2_^2^(12) ring motifs. The dimers are further connected by weak C—H⋯O hydrogen bonds into an infinite tape parallel to the *b* axis. In addition, π–π stacking inter­actions [centroid–centroid distance = 3.6569 (19) Å] and short inter­molecular contacts [O⋯O = 2.827 (3) and C⋯C = 3.088 (5) Å] are observed.

## Related literature

For the biological activity of sydnones, see: Rai *et al.* (2008 ▶); Hegde *et al.* (2008 ▶). For electrophilic substitution reaction on sydnones, see: Kalluraya & Rahiman (1997 ▶); Kalluraya *et al.* (2002 ▶). For hydrogen-bond motifs, see: Bernstein *et al.* (1995 ▶). For the stability of the temperature controller used in the data collection, see: Cosier & Glazer (1986 ▶).
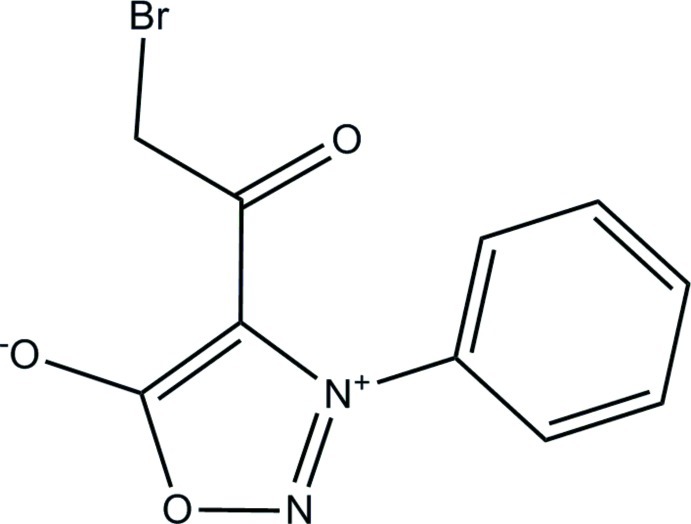


## Experimental

### 

#### Crystal data

C_10_H_7_BrN_2_O_3_
*M* *_r_* = 283.09
Monoclinic, 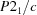

*a* = 7.2030 (2) Å
*b* = 5.8778 (1) Å
*c* = 25.1133 (5) Å
β = 91.104 (2)°
*V* = 1063.04 (4) Å^3^
*Z* = 4
Mo *K*α radiation
μ = 3.86 mm^−1^
*T* = 100 K
0.50 × 0.26 × 0.09 mm

#### Data collection

Bruker SMART APEXII CCD area-detector diffractometer
Absorption correction: multi-scan (*SADABS*; Bruker, 2009 ▶) *T*_min_ = 0.248, *T*_max_ = 0.720
11985 measured reflections
3710 independent reflections
3041 reflections with *I* > 2σ(*I*)
*R*_int_ = 0.035

#### Refinement

*R*[*F* ^2^ > 2σ(*F* ^2^)] = 0.059
*wR*(*F* ^2^) = 0.133
*S* = 1.23
3710 reflections
145 parameters
H-atom parameters constrained
Δρ_max_ = 0.95 e Å^−3^
Δρ_min_ = −0.96 e Å^−3^

### 

Data collection: *APEX2* (Bruker, 2009 ▶); cell refinement: *SAINT* (Bruker, 2009 ▶); data reduction: *SAINT*; program(s) used to solve structure: *SHELXTL* (Sheldrick, 2008 ▶); program(s) used to refine structure: *SHELXTL*; molecular graphics: *SHELXTL*; software used to prepare material for publication: *SHELXTL* and *PLATON* (Spek, 2009 ▶).

## Supplementary Material

Crystal structure: contains datablock(s) global, I. DOI: 10.1107/S1600536812026049/lh5486sup1.cif

Structure factors: contains datablock(s) I. DOI: 10.1107/S1600536812026049/lh5486Isup2.hkl

Supplementary material file. DOI: 10.1107/S1600536812026049/lh5486Isup3.cml

Additional supplementary materials:  crystallographic information; 3D view; checkCIF report

## Figures and Tables

**Table 1:** Hydrogen-bond geometry (Å, °)

*D*—H⋯*A*	*D*—H	H⋯*A*	*D*⋯*A*	*D*—H⋯*A*
C5—H5*A*⋯O3^i^	0.95	2.56	3.490 (5)	167
C10—H10*A*⋯O2^ii^	0.99	2.27	3.227 (5)	162

Symmetry codes: (i) 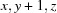; (ii) 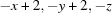.

**Fig. 1.**
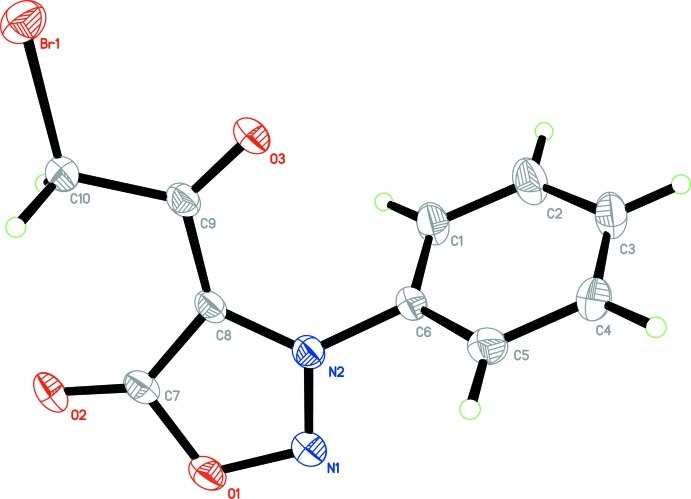
The molecular structure of the title compound with atom labels and 50% probability displacement ellipsoids.

**Fig. 2.**
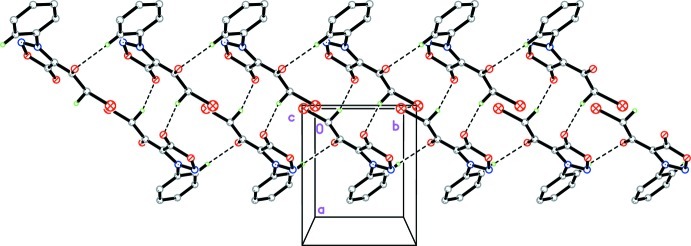
The crystal packing of the title compound. The dashed lines represent the hydrogen bonds. For clarity sake, hydrogen atoms not involved in hydrogen bonding have been omitted.

## supplementary crystallographic information

### Comment

Sydnones constitute a well-defined class of mesoionic compounds consisting of the 1,2,3-oxadiazole ring system. The study of sydnones remains a field of interest because of their electronic structure and also because of the varied types of biological activities displayed by some of them (Rai *et al.*, 2008). Sydnone derivatives were found to exhibit promising anti-microbial properties (Hegde *et al.*, 2008). Sydnones are synthesized by the cyclodehydration of *N*-nitroso-*N*-substituted amino acids using acetic anhydride. The sydnones unsubstituted in the 4-position readily undergo typical electrophilic substitution reaction namely formylation (Kalluraya & Rahiman, 1997) and acetylation (Kalluraya *et al.*, 2002).

The asymmetric unit of the title compound is shown in Fig. 1. The 1,2,3-oxadiazole ring (O1/N1/N2/C7/C8) and bromoacetyl group (Br1/O3/C9/C10) are almost planar [maximum deviation = 0.010 (4) and 0.013 (3) Å, respectively] and make dihedral angles of 59.31 (19) and 67.96 (11)°, respectively, with the C1–C6 benzene ring. The 1,2,3-oxadiazole ring is slightly twisted from the bromoacetyl group as indicated by the dihedral angle of 9.16 (24)°.

In the crystal (Fig. 2), molecules are linked by a pair of intermolecular C10—H10A···O2^ii^ hydrogen bonds (Table 1) into inversion dimers with an R_2_^2^(12) ring motif (Bernstein *et al.*, 1995). The dimers are further connected by intermolecular C5—H5A···O3^i^ hydrogen bonds (Table 1) into an infinite tape parallel to the *b* axis. The crystal is further stabilized by π···π interactions with a *Cg*1..*Cg*1 distance of 3.6569 (19) Å [symmetry code = 1-x,2-y,-z], where *Cg*1 is the centroid of O1/N1/N2/C7/C8 ring. Short intermolecular O1···O1(1-x, 3-y, -z) and C7···C7 (1-x, 2-y, -z) contacts of 2.827 (3) and 3.088 (5) Å, respectively, are also observed.

### Experimental

To a solution of 4-acetyl-3-arylsydnone (0.01 mol) in chloroform, bromine (0.01 mol) was added under visible light irradiation. The solvent was then removed under vacuum and the residue was recrystallized from ethanol. Yellow single crystals suitable for X-ray analysis were obtained by slow evaporation of an ethanol solution.

### Refinement

All H atoms were positioned geometrically [C—H = 0.95 and 0.99 Å] and refined using a riding model with *U*_iso_(H) = 1.2*U*_eq_(C).

### Crystal data

**Table:**

C_10_H_7_BrN_2_O_3_	*F*(000) = 560
*M**_r_* = 283.09	*D*_x_ = 1.769 Mg m^−^^3^
Monoclinic, *P*2_1_/*c*	Mo *K*α radiation, λ = 0.71073 Å
Hall symbol: -P 2ybc	Cell parameters from 4277 reflections
*a* = 7.2030 (2) Å	θ = 3.2–31.7°
*b* = 5.8778 (1) Å	µ = 3.86 mm^−^^1^
*c* = 25.1133 (5) Å	*T* = 100 K
β = 91.104 (2)°	Plate, yellow
*V* = 1063.04 (4) Å^3^	0.50 × 0.26 × 0.09 mm
*Z* = 4	

### Data collection

**Table:**

Bruker SMART APEXII CCD area-detector diffractometer	3710 independent reflections
Radiation source: fine-focus sealed tube	3041 reflections with *I* > 2σ(*I*)
Graphite monochromator	*R*_int_ = 0.035
φ and ω scans	θ_max_ = 32.1°, θ_min_ = 1.6°
Absorption correction: multi-scan (*SADABS*; Bruker, 2009)	*h* = −10→7
*T*_min_ = 0.248, *T*_max_ = 0.720	*k* = −8→8
11985 measured reflections	*l* = −37→36

### Refinement

**Table:**

Refinement on *F*^2^	Primary atom site location: structure-invariant direct methods
Least-squares matrix: full	Secondary atom site location: difference Fourier map
*R*[*F*^2^ > 2σ(*F*^2^)] = 0.059	Hydrogen site location: inferred from neighbouring sites
*wR*(*F*^2^) = 0.133	H-atom parameters constrained
*S* = 1.23	*w* = 1/[σ^2^(*F*_o_^2^) + (0.0334*P*)^2^ + 3.1942*P*] where *P* = (*F*_o_^2^ + 2*F*_c_^2^)/3
3710 reflections	(Δ/σ)_max_ < 0.001
145 parameters	Δρ_max_ = 0.95 e Å^−^^3^
0 restraints	Δρ_min_ = −0.96 e Å^−^^3^

### Special details

**Table:**

Experimental. The crystal was placed in the cold stream of an Oxford Cryosystems Cobra open-flow nitrogen cryostat (Cosier & Glazer, 1986) operating at 100.0 (1) K.
Geometry. All e.s.d.'s (except the e.s.d. in the dihedral angle between two l.s. planes) are estimated using the full covariance matrix. The cell e.s.d.'s are taken into account individually in the estimation of e.s.d.'s in distances, angles and torsion angles; correlations between e.s.d.'s in cell parameters are only used when they are defined by crystal symmetry. An approximate (isotropic) treatment of cell e.s.d.'s is used for estimating e.s.d.'s involving l.s. planes.
Refinement. Refinement of *F*^2^ against ALL reflections. The weighted *R*-factor *wR* and goodness of fit *S* are based on *F*^2^, conventional *R*-factors *R* are based on *F*, with *F* set to zero for negative *F*^2^. The threshold expression of *F*^2^ > σ(*F*^2^) is used only for calculating *R*-factors(gt) *etc*. and is not relevant to the choice of reflections for refinement. *R*-factors based on *F*^2^ are statistically about twice as large as those based on *F*, and *R*- factors based on ALL data will be even larger.

### Fractional atomic coordinates and isotropic or equivalent isotropic displacement parameters (Å^2^)

**Table:**

	*x*	*y*	*z*	*U*_iso_*/*U*_eq_	
Br1	0.99194 (5)	0.42975 (6)	0.098694 (16)	0.02954 (12)	
O1	0.5816 (4)	1.3028 (4)	0.02259 (10)	0.0249 (5)	
O2	0.7743 (4)	1.0452 (5)	−0.01567 (9)	0.0252 (5)	
O3	0.7030 (4)	0.7452 (5)	0.14346 (10)	0.0286 (6)	
N1	0.4819 (4)	1.3224 (5)	0.06815 (12)	0.0250 (6)	
N2	0.5218 (4)	1.1415 (5)	0.09563 (11)	0.0195 (5)	
C1	0.3074 (5)	0.9238 (7)	0.14915 (14)	0.0269 (7)	
H1A	0.2992	0.8127	0.1217	0.032*	
C2	0.2059 (6)	0.9026 (8)	0.19541 (16)	0.0322 (8)	
H2A	0.1261	0.7757	0.1999	0.039*	
C3	0.2213 (6)	1.0680 (8)	0.23529 (15)	0.0321 (8)	
H3A	0.1519	1.0519	0.2669	0.038*	
C4	0.3365 (6)	1.2553 (7)	0.22939 (15)	0.0305 (8)	
H4A	0.3455	1.3665	0.2568	0.037*	
C5	0.4388 (6)	1.2797 (6)	0.18323 (14)	0.0259 (7)	
H5A	0.5182	1.4069	0.1785	0.031*	
C6	0.4213 (5)	1.1132 (6)	0.14450 (13)	0.0213 (6)	
C7	0.6843 (5)	1.0981 (6)	0.02248 (13)	0.0215 (6)	
C8	0.6438 (5)	0.9965 (6)	0.07241 (13)	0.0187 (6)	
C9	0.7339 (5)	0.8024 (6)	0.09781 (13)	0.0203 (6)	
C10	0.8754 (5)	0.6855 (6)	0.06322 (14)	0.0240 (7)	
H10A	0.9721	0.7967	0.0534	0.029*	
H10B	0.8133	0.6322	0.0300	0.029*	

### Atomic displacement parameters (Å^2^)

**Table:**

	*U*^11^	*U*^22^	*U*^33^	*U*^12^	*U*^13^	*U*^23^
Br1	0.02838 (18)	0.02301 (17)	0.0372 (2)	0.00214 (16)	0.00027 (14)	−0.00141 (16)
O1	0.0304 (13)	0.0241 (12)	0.0203 (11)	−0.0022 (11)	0.0046 (10)	0.0053 (10)
O2	0.0260 (12)	0.0311 (14)	0.0188 (11)	−0.0056 (11)	0.0069 (9)	0.0033 (10)
O3	0.0391 (15)	0.0261 (13)	0.0210 (12)	0.0044 (11)	0.0120 (11)	0.0071 (10)
N1	0.0297 (15)	0.0222 (14)	0.0232 (14)	0.0000 (12)	0.0066 (12)	0.0026 (11)
N2	0.0209 (13)	0.0187 (12)	0.0190 (12)	−0.0024 (10)	0.0035 (10)	0.0001 (10)
C1	0.0294 (17)	0.0287 (17)	0.0230 (16)	−0.0062 (16)	0.0079 (13)	−0.0050 (14)
C2	0.0300 (18)	0.037 (2)	0.0297 (18)	−0.0072 (17)	0.0112 (15)	−0.0026 (16)
C3	0.0342 (19)	0.038 (2)	0.0242 (17)	0.0076 (18)	0.0109 (14)	−0.0005 (16)
C4	0.044 (2)	0.0258 (17)	0.0221 (17)	0.0039 (16)	0.0078 (15)	−0.0050 (14)
C5	0.0344 (18)	0.0199 (15)	0.0236 (16)	−0.0016 (14)	0.0036 (14)	−0.0001 (13)
C6	0.0269 (16)	0.0197 (15)	0.0174 (14)	0.0003 (12)	0.0062 (12)	0.0005 (11)
C7	0.0229 (14)	0.0209 (15)	0.0208 (15)	−0.0053 (12)	0.0030 (12)	0.0028 (12)
C8	0.0213 (14)	0.0178 (13)	0.0172 (14)	−0.0053 (12)	0.0049 (11)	0.0008 (11)
C9	0.0229 (15)	0.0180 (14)	0.0201 (14)	−0.0044 (12)	0.0054 (12)	0.0008 (12)
C10	0.0258 (16)	0.0197 (15)	0.0269 (17)	0.0006 (13)	0.0091 (13)	0.0026 (13)

### Geometric parameters (Å, º)

**Table:**

Br1—C10	1.931 (4)	C2—H2A	0.9500
O1—N1	1.368 (4)	C3—C4	1.388 (6)
O1—C7	1.412 (4)	C3—H3A	0.9500
O2—C7	1.208 (4)	C4—C5	1.393 (5)
O3—C9	1.219 (4)	C4—H4A	0.9500
N1—N2	1.297 (4)	C5—C6	1.384 (5)
N2—C8	1.363 (4)	C5—H5A	0.9500
N2—C6	1.446 (4)	C7—C8	1.424 (4)
C1—C6	1.390 (5)	C8—C9	1.454 (5)
C1—C2	1.390 (5)	C9—C10	1.517 (5)
C1—H1A	0.9500	C10—H10A	0.9900
C2—C3	1.399 (6)	C10—H10B	0.9900
			
N1—O1—C7	110.9 (3)	C4—C5—H5A	121.0
N2—N1—O1	105.2 (3)	C5—C6—C1	123.6 (3)
N1—N2—C8	115.0 (3)	C5—C6—N2	118.4 (3)
N1—N2—C6	115.9 (3)	C1—C6—N2	118.0 (3)
C8—N2—C6	128.9 (3)	O2—C7—O1	120.7 (3)
C6—C1—C2	117.6 (3)	O2—C7—C8	135.4 (3)
C6—C1—H1A	121.2	O1—C7—C8	103.9 (3)
C2—C1—H1A	121.2	N2—C8—C7	105.0 (3)
C1—C2—C3	120.1 (4)	N2—C8—C9	126.1 (3)
C1—C2—H2A	120.0	C7—C8—C9	128.2 (3)
C3—C2—H2A	120.0	O3—C9—C8	122.7 (3)
C4—C3—C2	120.9 (3)	O3—C9—C10	123.4 (3)
C4—C3—H3A	119.5	C8—C9—C10	113.8 (3)
C2—C3—H3A	119.5	C9—C10—Br1	112.3 (2)
C3—C4—C5	119.8 (3)	C9—C10—H10A	109.1
C3—C4—H4A	120.1	Br1—C10—H10A	109.1
C5—C4—H4A	120.1	C9—C10—H10B	109.1
C6—C5—C4	118.0 (3)	Br1—C10—H10B	109.1
C6—C5—H5A	121.0	H10A—C10—H10B	107.9
			
C7—O1—N1—N2	0.9 (4)	N1—O1—C7—C8	−1.7 (4)
O1—N1—N2—C8	0.3 (4)	N1—N2—C8—C7	−1.3 (4)
O1—N1—N2—C6	−175.4 (3)	C6—N2—C8—C7	173.7 (3)
C6—C1—C2—C3	−0.3 (6)	N1—N2—C8—C9	169.6 (3)
C1—C2—C3—C4	0.3 (7)	C6—N2—C8—C9	−15.4 (6)
C2—C3—C4—C5	−0.1 (6)	O2—C7—C8—N2	−176.0 (4)
C3—C4—C5—C6	−0.1 (6)	O1—C7—C8—N2	1.7 (3)
C4—C5—C6—C1	0.1 (6)	O2—C7—C8—C9	13.4 (7)
C4—C5—C6—N2	177.8 (3)	O1—C7—C8—C9	−168.9 (3)
C2—C1—C6—C5	0.1 (6)	N2—C8—C9—O3	2.4 (6)
C2—C1—C6—N2	−177.6 (4)	C7—C8—C9—O3	171.2 (4)
N1—N2—C6—C5	−59.9 (4)	N2—C8—C9—C10	−174.9 (3)
C8—N2—C6—C5	125.1 (4)	C7—C8—C9—C10	−6.2 (5)
N1—N2—C6—C1	117.9 (4)	O3—C9—C10—Br1	2.4 (5)
C8—N2—C6—C1	−57.1 (5)	C8—C9—C10—Br1	179.8 (2)
N1—O1—C7—O2	176.4 (3)		

### Hydrogen-bond geometry (Å, º)

**Table:**

*D*—H···*A*	*D*—H	H···*A*	*D*···*A*	*D*—H···*A*
C5—H5*A*···O3^i^	0.95	2.56	3.490 (5)	167
C10—H10*A*···O2^ii^	0.99	2.27	3.227 (5)	162

Symmetry codes: (i) *x*, *y*+1, *z*; (ii) −*x*+2, −*y*+2, −*z*.
